# Brazilian Protocol for Sexually Transmitted Infections 2020: epidemiological surveillance

**DOI:** 10.1590/0037-8682-549-2020

**Published:** 2021-05-17

**Authors:** Carmen Silvia Bruniera Domingues, Leonor Henriette de Lannoy, Valeria Saraceni, Alessandro Ricardo Caruso da Cunha, Gerson Fernando Mendes Pereira

**Affiliations:** 1 Secretaria de Estado da Saúde de São Paulo, Centro de Referência e Treinamento de Doenças Sexualmente Transmissíveis e Aids, São Paulo, SP, Brazil.; 2 Ministério da Saúde, Secretaria de Vigilância em Saúde, Brasilia, DF, Brazil.; 3 Secretaria Municipal de Saúde do Rio de Janeiro, Rio de Janeiro, RJ, Brazil.

**Keywords:** Public health surveillance, Health information systems, Syphilis, Congenital, Disease notification, Epidemiological monitoring

## Abstract

This article summarizes the chapter on epidemiological surveillance of sexually transmitted infections (STI) that comprises the 2020 Clinical Protocol and Therapeutic Guidelines (PCDT) for Comprehensive Care for People with STI, published by the Health Surveillance Department of the Brazilian Ministry of Health. It presents some reflections on the new case definitions for surveillance of acquired syphilis, syphilis in pregnant women, and congenital syphilis. The 2020 PCDT-IST was elaborated grounded on scientific evidence and validated in discussions with specialists. Epidemiological and clinical aspects are addressed, and health service managers' guidelines regarding programmatic and operational management of these diseases are presented. Guidelines for health professionals on screening, diagnosing, and treating people with STI and their sex partners, in addition to strategies for surveillance, prevention, and control actions, are also published.

## Highlighted excerpt

sexually transmitted infections are among the most common diseases in the world. They affect people's health and life, impact reproductive and child health, contribute to infertility and complications during pregnancy and birth.

## FOREWORD

This article summarizes the chapter on epidemiological surveillance of sexually transmitted infections (STI) that composes the Clinical Protocol and Therapeutic Guidelines (PCDT) for Comprehensive Care for People with Sexually Transmitted Infections (STI), published by the Health Surveillance Secretariat of the Ministry of Health. For elaborating the PCDT, a selection and analysis of the evidence available in the literature were performed, and a panel of specialists discussed it. The document was approved by the National Committee for Technology Incorporation into the Brazilian National Health System (Conitec)^1^, and was updated by the team of specialists in STI of the 2020 PCDT-IST^2^.

## INTRODUCTION

STI are caused by a virus, bacteria, and other microorganisms, transmitted mainly in condomless sexual contact². They are considered a public health issue, among the most common diseases in the world. They affect people's health and life, cause a significant impact on reproductive and child health, contribute to infertility, complications of pregnancy and birth, and make the human immunodeficiency virus (HIV) transmission easier, and in extreme cases, can cause fetal death^3^.

In Brazil, the bases for STI prevention, diagnosis, and treatment are well-established, and its epidemiological surveillance model comprises compulsory notification, sentinel services, and transversal studies in certain population groups^4^.

**FIGURE 1:**
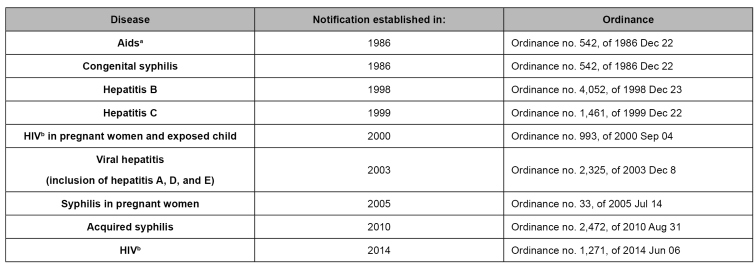
Ordinances that established the compulsory notification of sexually transmitted infections and congenital syphilis, Brazil.

^a^Aids: acquired immunodeficiency syndrome; ^b^HIV: human immunodeficiency virus.

STIs that are part of the national compulsory notification list^5^ ^,^ ^6^include the acquired immunodeficiency syndrome, HIV, HIV in pregnant women, viral hepatitis, syphilis in pregnant women, acquired syphilis, and male urethral discharge syndrome cases^7^ (Figure 1). The protocols for HIV and hepatitis are specific and treated separately^8^ ^-^ ^11^. Throughout Brazil, congenital syphilis, syphilis in pregnant women, and acquired syphilis compulsory notification started in 1986, 2005, and 2010 respectively (Figure 1).

Suspected or confirmed cases on the national compulsory notification list are notified through the Notifiable Diseases Information System (Sinan) and follow an upward flow, starting at municipal, through states, to national surveillance. At each stage of the process, the different encompassed players are responsible for collecting, analyzing, and disclosing the information generated by the National Epidemiological Surveillance System, contributing to the improvement of the health situation, supporting the planning and the adoption of control measures needed for the whole or specific groups of the Brazilian population, as well as for monitoring and assessing health policies, plans, and programs^12^.

This article aims at presenting some reflections on the new case definitions for the acquired syphilis, syphilis in pregnant women, and congenital syphilis surveillance, according to the 2020 PCDT for Comprehensive Care for People with STI^2^.

## EPIDEMIOLOGICAL ASPECTS

Based on prevalence data for the period 2009-2016, the World Health Organization (WHO) estimated a total of 376.4 million STI considered curable cases. From this total, 127.2 million (95% CI 95.1;165.9) were estimated chlamydia cases, 86.9 million (95% CI58.6;123.4) of gonorrhea, 156.0 million (95% CI 103.4;231.2) of trichomonas infections, and 6.3 million (95% CI 5.5;7.1) of syphilis. The estimated global syphilis prevalence in men and women was 0.5% (95% CI 0.4;0.6), with regional values ranging from 0.1 to 1.6%^13^.

In Brazil, syphilis prevalence can be estimated through transversal studies conducted in specific populations^14^ ^-^ ^19^. Parturient women have been a target for such studies, monitored for presenting prevalence similar to that of the general female population. The national estimates range from 1.7% (95% CI 1.2;2.2), in 2000, to 1.02% (95% CI 0.84;1.25) in 2011-2012^14^ ^-^ ^17^. Among the conscripted Brazilian adolescents, the prevalence of syphilis was estimated for the last time in 2016, reaching 1.1% (CI 95% 0.85;1.40)^18^. The active syphilis estimate in female sex workers ranged from 2.4% (CI 95% 1.7;3.4), in 2009, to 8.5% (CI 95% 7.3;9.7) in 2016^19^.

**FIGURE 2:**
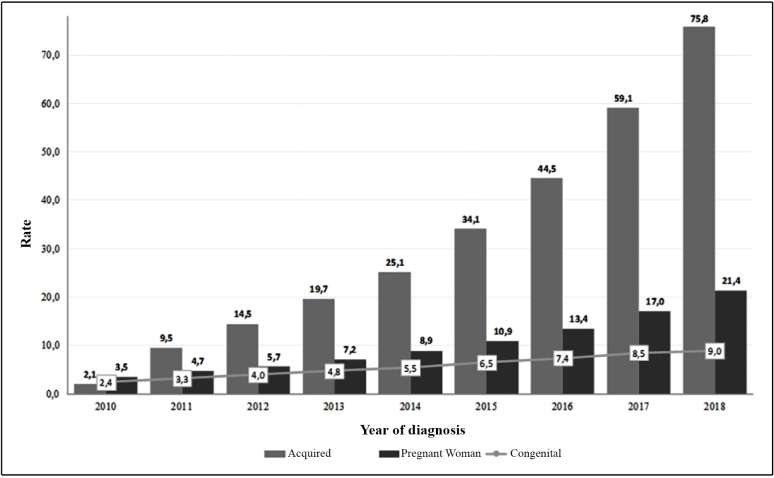
Acquired syphilis detection rate (per 100,000 inhabitants), syphilis in pregnant women detection rate, and congenital syphilis incidence rate (per 1,000 live births) by year of diagnosis, Brazil, 2010-2018.

**FIGURE 3:**
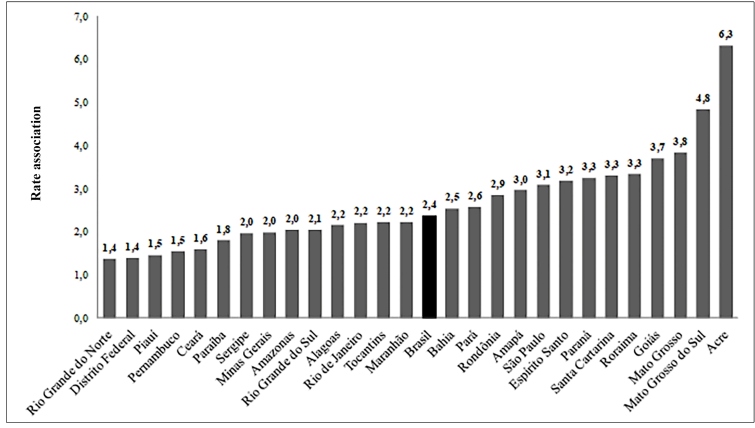
Syphilis in pregnant women detection rate and congenital syphilis incidence rate association by states, Brazil, 2018.

The growing number of cases has indicated the persistence of these diseases, which, although avoidable, continue to challenge the health systems. Brazil registered 650,258 acquired syphilis cases for the period 2010 to June 30, 2019, 324,321 cases of syphilis in pregnant women for the period 2005 to June 30, 2019, and 214,891 of congenital syphilis for the period 1998 to June 30, 2019^20^.In the period from 2014 to 2018 (Figure 2), the acquired syphilis detection rate increased three times (from 25.1 to 75.8 cases per 100,000 inhabitants); that of syphilis in pregnant women, 2.4 times (from 8.9 to 21.4 cases per 1,000 live births); and the congenital syphilis incidence rate, in 1.6 times (from 5.5 to 9.0 cases per 1,000 live births). In 2018, the association between the detection rates of syphilis in pregnant women and congenital syphilis incidence was 2.4 pregnant women with syphilis for one child with congenital syphilis, and in 14 states, this association is below the Brazilian national level (Figure 3). Congenital syphilis incidence still presents values much higher than those established by WHO for eliminating this disease^21^.

**Source:***Sistema de Informação de Agravos de Notificação* (Sinam) - Brazil.

**Source:***Sistema de Informação de Agravos de Notificação* (Sinam) - Brazil.

Despite the high detection of cases, it is known that syphilis is an underreported disease^22^ ^,^ ^23^, with implications for the response to STI in Brazil, considering the total number of cases and aspects associated with behavior and vulnerability. Case underestimation can harm the health system planning regarding continuous input provision and priority actions implementation, primarily those addressed to the most vulnerable populations^2^.

In 2017, to be aligned to the Pan American Health Organization (PAHO) recommendations, the Ministry of Health updated the definition of acquired syphilis, syphilis in pregnant women, and congenital syphilis cases through Informative Notice no. 2 - SEI/2017 - DIAHV/SVS/MS^24^. However, the electronic format of Sinan was not adapted to the forms' changes, which caused some difficulties in aligning the criteria at the health service level. The case definition must be clear, objective and well understood, as it establishes a standard that allows for a set of criteria deciding if a person presents or not a particular infection or disease, making the cases comparable in the registry on the surveillance system^25^ ^,^ ^26^.

**FIGURE 4:**
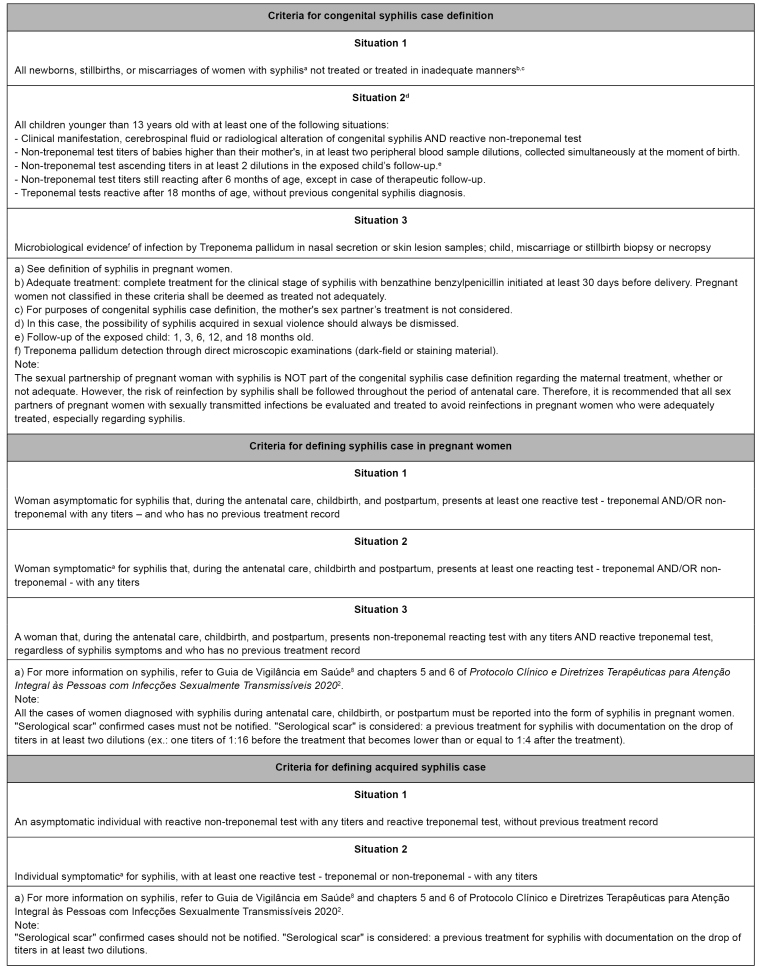
Criteria for congenital syphilis, syphilis in pregnant women, and acquired syphilis case definition, Brazil.

The definitions of acquired syphilis, syphilis in pregnant women, and congenital syphilis are presented below (Figure 4).

## ACQUIRED SYPHILIS

Two case definition criteria were established for epidemiological surveillance purposes, called Situation 1 and Situation 2, which allow acquired syphilis to be reported. The case definition is comprehensive, allowing individuals with syphilis in any clinical phase of the disease, primarily when classified by the first criterion or Situation 1 (Figure 4). 

Acquired syphilis has a notification and investigation form that can be used by municipal and state epidemiological surveillance. On Sinan, only data regarding case notification is inserted, that is, identification and demographic data of the affected person. To better understand the disease, variables corresponding to the epidemiological investigation component, such as clinical and laboratory data, behavior, and vulnerability, should also be included in the system.

It is important to provide information on the clinical phase of the disease, types of diagnostic tests (treponemal and non-treponemal test), treatment conducted (benzathine benzylpenicillin or another drug), and any other elements that can contribute to the epidemiological analysis, as well as verify compliance with the case definition established in Brazil. It is also essential to differentiate case duplicity and reinfections - when the same individual is notified multiple times. 

In Brazil, the acquired syphilis profile is linked to the health services' ability to detect and report cases and their demographical characteristics such as sex, age, race or skin color, education, and residence. The data may refer to prevalent or incident cases since it is impossible to define the disease's clinical phase. It would be essential to identify incident cases, classified as recent syphilis, in recent, primary, secondary, and latent clinical form (up to one year of infection), a period considered greater transmissibility of the infectious agent, *Treponema pallidum* ^2^.

## SYPHILIS IN PREGNANT WOMEN

The latest case definition of syphilis in pregnant women, characterized by three situations, is more sensible and comprehensive. It includes women diagnosed at the moment of birth or postpartum, besides the pregnancy, contributing to expanding the disease's detection in the pregnancy-puerperal cycle (Figure 4). However, the notifications of parturient and postpartum women diagnosed with this disease must be conducted using the notification form for syphilis in pregnant women, whose investigation variables are associated, in most of the cases, with the data obtained during antenatal care. 

**FIGURE 5:**
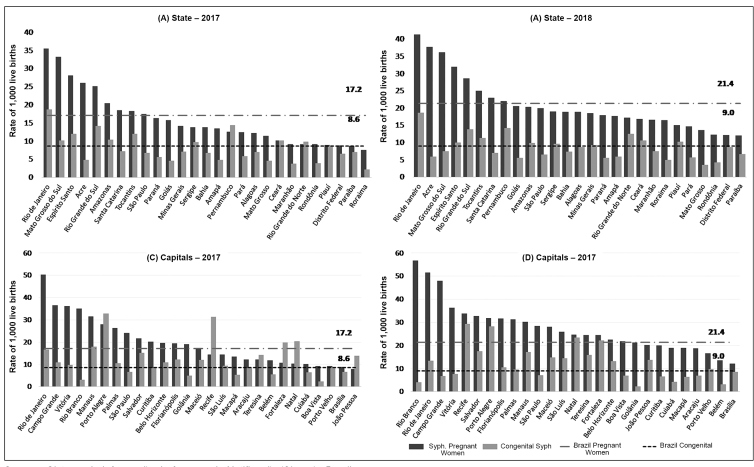
Syphilis in pregnant women detection rate and congenital syphilis incidence rate (per 1,000 live births) by states (UF) and capitals. Brazil, 2017-2018.

The increase in pregnant women with syphilis notifications, primarily when the detection is performed at the moment of birth or postpartum, may not reduce congenital syphilis due to missing maternal treatment opportunities. In 2017, when the pregnant women notification was exclusively conducted in antenatal care, it was possible to observe congenital syphilis incidence rates higher than those for syphilis detection in pregnant women in some Brazilian states and capitals^20^. From 2018, with the cases diagnosed in the birth or postpartum inclusion, syphilis in the pregnant women detection rate presented an increase, although it did not significantly impact congenital syphilis reduction (Figure 5).

**Source:***Sistema de Informação de Agravos de Notificação* (Sinam) - Brazil.

**2017:** Reported cases of pregnant women with syphilis during the antenatal care.

**2018:** Reported cases in antenatal care, childbirth, or postpartum.

In the case classification for Situation 1, a pregnant woman may be undue, with conflicting test results, mainly when the non-treponemal test presents a reaction and the treponemal test does not. Multiple times, professionals making the notification do not remember to consider the possibility of a false-positive result in the non-treponemal test and, consequently, the need for performing another treponemal test with a different methodology^2^. Nevertheless, this definition is very sensible and allows for the inclusion of cases when the service does not have both types of tests. 

In Situation 3, although the footnote highlights that confirmed "serological scar" cases shall not be informed to the surveillance system, unnecessary notifications or underreporting are also possible to occur, depending on the understanding of professionals. The concept of "serological scar" is described in PCDT-IST 2020 to provide better information on the meaning of "previous treatment for syphilis"^2^.

A criteria review for syphilis in pregnant women case definition, with attention to not losing sensitivity, could contribute to the notified cases' quality. It is possible that a case definition with only two criteria, therefore simpler, could be easy to understand for epidemiological surveillance services.

## CONGENITAL SYPHILIS

Since became a mandatory notification in 1986, congenital syphilis has its case definition periodically reviewed. Its last update, published at the end of 2017, established three situations for case definition (Figure 4). One of the main changes was excluding the sex partner’s treatment from the description of adequate maternal treatment, contributing to the Brazilian case definition becoming more specific and aligned with international concepts.

In the previous versions, the case criteria aimed to highlight sensitivity to include the highest possible occurrences. They were important, especially when Brazil presented a case underreporting and invisibility of congenital syphilis^27^. It is probable that the maternal sex partner’s exclusion, acting indirectly on the case definition of congenital syphilis, has impacted the infection incidence rate deceleration. For the period 2016-2017, a 15% increase of this rate was observed, from 7.4 to 8.5 cases per 1,000 live births, respectively (Figure 2), while for the period 2017-2018, when the new case definition was introduced, the incidence rate increase was 6%, from 8.5 to 9.0 cases per 1,000 live births^20^.

Congenital syphilis’ largest case portion is classified as Situation 1, and the smallest as Situation 3. The performance of child, miscarriage, or stillborn necropsy and the material collection from a skin lesion or nasal secretion for *Treponema pallidum* detection in direct microscopic examinations are limited diagnoses in the hospital or outpatient services^20^.

Regarding Situation 2, it is observed that the surveillance professionals show more difficulty in case of classification as per the following components: (i) rising titers of non-treponemal tests in at least two dilutions in the follow-up of the exposed child; (ii) titers of non-treponemal still reacting after six months of age, except in the situation of therapeutic follow-up; and (iii) treponemal tests reacting after 18 months of age, without a previous diagnosis of congenital syphilis. These criteria define the congenital syphilis case when the child is considered exposed to syphilis in the maternity ward and presents serological testing alterations during the outpatient follow-up. They are also used for children older than 18 months, without a previous congenital syphilis diagnosis or exposure to maternal syphilis. It should be highlighted that the possibility of syphilis acquired in child sexual abuse should be excluded^2^.

Qualified health care personnel in the Primary Health Care service is essential to the optimal management of congenital syphilis cases. Children exposed to maternal syphilis, but not considered active cases, should be linked to the primary care, in order to avoid missing diagnostic opportunities, treatment and underreporting of congenital syphilis cases detected after discharge from the maternity ward^28^ ^,^ ^29^.

Efforts to meet the goal of eliminating congenital syphilis as proposed by PAHO/WHO - 0.5 case per one thousand live births (including stillbirths)^21^, are needed, as, despite the change in case definition, making it more specific, the occurrence number keeps growing^20^, contributing to the incident rate increase to values much higher than the expected for its elimination (Figure 2). 

## REMARKS ON STI SURVEILLANCE, PREVENTION, AND CONTROL

In the considerations about STI surveillance, it is noted that in 2020, there still is no official data produced systematically on gonorrhea, chlamydia, trichomonas infections, or herpes genitalis in Brazil, as these are STI that are not part of the national notifiable disease list^6^. However, it is possible to estimate their prevalence with study development on certain populations, assisted by specific services. 

The Ministry of Health established two large projects for STI: "Rapid Response to Syphilis", aiming at reducing the acquired syphilis and syphilis in pregnant women occurrence, and eliminating congenital syphilis^30^; and "Sexually Transmitted Infections: Urethritides and Genital Ulcers Etiology Surveillance in Brazil and Analysis of Resistance to Antimicrobial Drugs", named project SenGono, aiming at monitoring the antimicrobial resistance of *Neisseria gonorrhoeae* strains present in the country, identifying the main etiological agents of the urethral discharge and genital ulcers, using molecular tests in specific services^30^ ^,^ ^31^.

Important information sources, in addition to Sinan, can be accessed routinely, through the municipal, state, and federal epidemiological surveillance services, to obtain supplementary information on STI, e.g., the Mortality Information System (SIM), Life Birth Information System (Sinasc), Hospital Information System (SIH) and Outpatient Information System (SIA), and the systems for performing the supplementary examinations^32^.

STI prevention is still a challenge. The lack of knowledge or perception of Brazil's syphilis situation and its social determinants, the health system’s weak points, especially regarding access and quality of prenatal care, are conditions contributing to congenital persistence of syphilis^33^ ^,^ ^34^. It is essential to maintain the political compromise with public health at all management levels, prioritizing prevention, early diagnosis, and the timely treatment of syphilis, the most effective strategy to eliminate congenital syphilis from Brazil^35^.

The health information systems, especially Sinan, are critical tools for surveillance actions^36^. It is vital to update and align the reviews of case definition, the epidemiological notification, the investigation form, and the information system that shall receive and preserve the database. According to the criteria established for the case definitions, the quality of the information shall improve as the data are systematically collected, inserted, and processed in the systems.

The acquired syphilis, syphilis in pregnant women, and congenital syphilis case definitions, based on sufficiently sensitive and more specific criteria, allow comparing the Brazilian epidemiological profile with those of other countries. Likewise, they produce information for the surveillance to monitor the disease’s behavior and trends, aiming at recommending prevention and control measures, in addition to intensifying actions for antenatal care, in order to stop the syphilis vertical transmission chain. 
